# Diagnostic Challenge of Tuberculous Pleural Effusion: Elevated Adenosine Deaminase (ADA) as the Key Indicator in the Absence of Microbiological Confirmation

**DOI:** 10.7759/cureus.93944

**Published:** 2025-10-06

**Authors:** Mikqdad A Alsaeed, Muhammad Farooq Abdul Sattar

**Affiliations:** 1 Internal Medicine Department, King Saud Medical City, Riyadh, SAU

**Keywords:** adenosine deaminase, intrapleural fibrinolysis, pleural biopsy, thoracoscopy, tuberculous pleural effusion

## Abstract

Tuberculous pleural effusion (TPE) is frequently paucibacillary. Consequently, acid-fast bacilli (AFB) smears, polymerase chain reaction (PCR), and mycobacterial cultures from pleural fluid are often negative. Pleural adenosine deaminase (ADA) is an important adjunct, but interpretation requires an appropriate clinical context. A previously healthy 20-year-old man presented with pleuritic chest pain, dyspnea, fever, and bilateral pleural effusions. Despite repeated negative microbiology for *Mycobacterium tuberculosis* (AFB smear, PCR, and cultures from sputum, bronchoalveolar lavage, pleural fluid, and pleural tissue) and a non-diagnostic pleural biopsy, pleural fluid was a lymphocyte-predominant exudate with markedly elevated ADA (126.5 and 100.0 U/L). The course was complicated by respiratory failure requiring intubation, surgical tracheostomy, video-assisted thoracoscopic surgery (VATS) decortication with talc pleurodesis, ventilator-associated pneumonia due to *Pseudomonas aeruginosa*, and only partial response to intrapleural alteplase for persistent loculations. Empirical first-line anti-tuberculous therapy (isoniazid, rifampin, pyrazinamide, ethambutol) was commenced on hospital day 66. Within 10 days, the patient showed improvement, pleural drainage ceased, oxygenation improved, and he was weaned from mechanical ventilation. He was discharged clinically stable after 117 days of hospitalization (on day 52 of anti-TB therapy). In high probability scenarios with lymphocytic exudate with high ADA and compatible clinical features, empirical anti-TB therapy can be justified despite repeatedly negative microbiology, particularly when alternative diagnoses are excluded and the patient fails to respond to appropriate antibiotics.

## Introduction

Tuberculous pleural effusion (TPE) is the second most common form of extrapulmonary tuberculosis (EPTB) after tuberculous lymphadenitis, accounting for approximately 24.1% of all EPTB cases [1]. The global burden of TB remains substantial, with an estimated 10.8 million new cases and 1.25 million deaths annually [2]. Despite advances in diagnosis and treatment, TPE continues to pose significant diagnostic challenges. TPE typically results from a delayed hypersensitivity reaction to *Mycobacterium tuberculosis *antigens in the pleural space or from the rupture of a subpleural focus. Patients commonly present with nonspecific symptoms, such as fever, pleuritic chest pain, and dyspnea. Diagnosis can be challenging due to the limited diagnostic yield of pleural fluid microbiological studies. While pleural biopsy has higher diagnostic sensitivity, it is invasive and not always feasible. A high adenosine deaminase (ADA) measurement in pleural fluid has emerged as a valuable diagnostic tool [3]. However, ADA can be falsely low in early disease, elderly patients, or in certain atypical presentations, leading to misdiagnosis or delayed treatment [4,5]. Moreover, unusual manifestations, such as bilateral effusions, hydropneumothorax, or chylothorax, further complicate clinical evaluation. We report a rare case of TPE with only evidence of high ADA and negative AFB smear, TB polymerase chain reaction (PCR), and *M. tuberculosis *culture, highlighting the diagnostic complexity and the need for a high index of suspicion even when conventional tests are inconclusive.

## Case presentation

**Figure 1:**
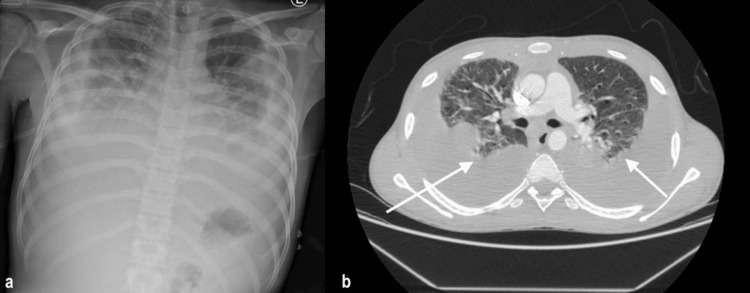
Initial images on presentation (a). X-ray showing massive bilateral effusion. (b) CT scan showing massive bilateral effusion with atelectatic changes bilaterally (white arrow).

A 20-year-old Ethiopian male, with no known medical history or TB exposure, presented with a one-week history of left-sided pleuritic chest pain, progressive shortness of breath, dry cough, anorexia, and weight loss. On arrival, he was tachypneic, hypoxic (SpO₂ of 86% on room air), and febrile (temp of 38.9°C). Chest examination revealed decreased air entry bilaterally, more prominent on the left. Chest radiograph and CT imaging showed moderate-to-large bilateral pleural effusions and bilateral compressive atelectasis (Figure 1). Initial systemic laboratory (Table 1) showed high inflammatory markers.

**Table 1:** Systemic laboratory findings CRP: C-reactive protein; ESR: erythrocyte sedimentation rate; MCV: mean corpuscular volume; MCH: mean corpuscular hemoglobin; LDH: lactate dehydrogenase

Parameter	Result	Reference Range/Notes
WBC	4.1 × 10³/µL	4.0–11.0
Hemoglobin	10.1 g/dL	12–16
MCV	82 fL	80–96
MCH	24.8 pg	27–32
Platelets	281 × 10³/µL	150–400
Neutrophils (Abs)	3.09 × 10³/µL	2.0–7.0
Lymphocytes (Abs)	0.34 × 10³/µL	1.0–3.0
ESR	56 mm/h	<20
CRP	125.8 mg/L	<5
Serum LDH	210 U/L	<250 U/L
Serum Protein	6.5 g/dL	6.0–8.0 g/dL

**Figure 2:**
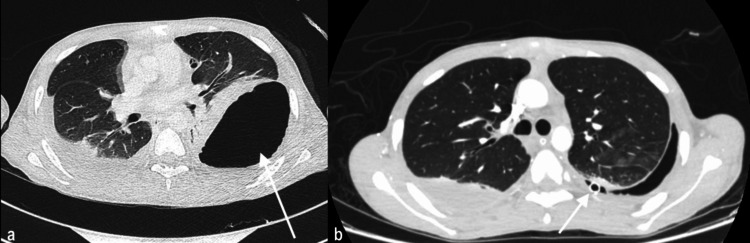
Follow-up CT scan (a). Development iatrogenic pneumothorax post malposition of ICT with bilateral pleural effusion. (b). Improvement of the pneumothorax with pleural effusion post reinsertion of ICT.

He was admitted with a working diagnosis of severe parapneumonic effusion and was started empirically on piperacillin-tazobactam and azithromycin. Bilateral intercostal chest tubes (ICT) were initially placed. The left-side ICT was malpositioned into the subcutaneous tissue, leading to a localized iatrogenic hydropneumothorax. This was confirmed by CT and promptly revised, with a new ICT inserted correctly and yielding over 1,000 mL of turbid yellow effusion (Figure 2).

Due to clinical deterioration, the patient was intubated. Serial pleural fluid analysis revealed (Table 2) a lymphocyte-predominant exudate with markedly elevated ADA levels (126.5 U/L and 100.0 U/L; reference range: <9.2), high LDH (>4,000 IU/L), and negative cytology. However, all microbiological studies - including multiple AFB smears, TB PCRs, and *M. tuberculosis *cultures from sputum, pleural fluid, bronchoalveolar lavage, and pleural tissue - were negative. The patient's clinical condition remained critical with no improvement in oxygen requirements; his FiO₂was 100%. Therefore, surgical tracheostomy was performed for prolonged ventilation. 

**Table 2:** Pleural fluid analyses (serial samples) Pleural fluid consistently demonstrated an exudative profile by Light’s criteria. The first sample showed lymphocytic predominance (59%), which is typical of tuberculous pleurisy. Subsequent samples were more neutrophil-rich, reflecting secondary inflammatory response. Na: data not availabl; ADA: adenosine deaminase; LDH: lactate dehydrogenase

Sample	WBC (×10³/µL)	RBC (×10⁶/µL)	PMN %	MN %	Color	Appearance	ADA (U/L)	LDH (U/L)	Protein (g/dL)
1	6.7	0.000	41.2	58.8	Yellow	Turbid	126.5	580	4.8
2	7.0	0.011	82.8	17.2	Yellow	Turbid	100	493	4.6
3	128.0	0.010	65.7	34.3	Yellow	Hazy	Na	Na	Na

Furthermore, video-assisted thoracoscopic surgery (VATS) was performed with pleural decortication, biopsy, and talc pleurodesis. Intraoperative findings revealed dense adhesions, thickened pleura, and multiloculated empyema. Histopathology showed granulation tissue with acute and chronic inflammation, without granuloma or malignancy. AFB stain, TB PCR, and *M. tuberculosis *culture on tissue were again negative.

Postoperatively, he developed ventilator-associated pneumonia (VAP) with *Pseudomonas aeruginosa *isolated from tracheal aspirate, pleural fluid, and pleural tissue cultures. Although therapy was adjusted according to sensitivities, and despite broad-spectrum antimicrobials - piperacillin-tazobactam, azithromycin, vancomycin, and meropenem - he showed no clinical or radiological improvement.

Serial CT scans documented persistent bilateral effusions, worsening loculations, and pleural thickening. His condition did not improve, drainage from pleural fluids persisted, and pleural collections remained unresolved; thus, a trial of intrapleural thrombolytics (alteplase) was initiated over three doses across six days, with only partial improvement.

Nevertheless, the persistently elevated ADA, lymphocytic exudate, lack of alternative diagnosis, and failure to respond to antibiotics or thrombolytics supported a presumptive diagnosis of TPE.

On day 66 of admission, empirical first-line anti-tuberculous therapy (isoniazid, rifampin, pyrazinamide, ethambutol) was initiated. Within 10 days, the patient showed dramatic improvement, including resolution of fever, reduced pleural output until it reached nil, and improved oxygenation. He was extubated and kept on tracheostomy, which was decannulated one week before discharge. The patient was discharged after 117 days of hospitalization, on day 52 of anti-TB therapy, clinically stable, ambulatory, and with arrangements for outpatient follow-up.

## Discussion

TPE remains a diagnostic challenge due to its paucibacillary nature and the low yield of microbiological confirmation. Acid-fast bacilli (AFB) smears from pleural fluid are rarely positive, with reported positivity rates of less than 5%. Similarly, TB PCR, and *M. tuberculosis *culture demonstrate limited sensitivity, only positive in 33.3% and 34.8% of cases, respectively [6,7].

In our case, all TB microbiologic workups - including sputum, pleural fluid, bronchoalveolar lavage (BAL), and pleural biopsy - were negative, highlighting the diagnostic limitations in real-world settings. Moreover, an eight-year retrospective review of 103 pleural TB cases, in which only 15.5% had microbiologically confirmed diagnoses, highlights the reliance on indirect diagnostic markers in routine practice [8].

Given these constraints, pleural fluid ADA has emerged as a valuable adjunct. ADA levels ≥40 IU/L are associated with high sensitivity (93%) and specificity (90%) for TPE, especially in TB-endemic areas [9]. Although the optimal cut-off remains debated, ADA values >70 IU/L are strongly suggestive of TPE [10]. In our patient, ADA was markedly elevated at 126.5 U/L and 100.0 U/L, with lymphocyte-predominant exudate, particularly when other causes, such as lymphoma, empyema, or autoimmune conditions, were excluded via cytology, histopathology, and autoantibody panels, which further supported the presumptive diagnosis of TPE.

The clinical course of our patient was complicated and protracted. He presented with bilateral pleural effusions, significant hypoxia, and rapidly progressed to requiring mechanical ventilation. Initial empirical antibiotics failed to produce clinical improvement. In addition, thoracic imaging showed compressive atelectasis and loculated hydropneumothorax, which prompted thoracic surgical intervention. VATS with decortication and pleurodesis revealed thickened pleura and multiloculated empyema, but the pleural biopsy did not show granulomas or malignancy.

Intrapleural fibrinolysis using alteplase was attempted for unresolved effusions, as guided by the MIST-2 trial, which supports their use in complicated parapneumonic effusions [11]. However, its role in TPE remains controversial, and our patient showed only a partial response. Literature suggests that, in loculated TPEs, fibrinolytics may accelerate effusion resolution and reduce long-term pleural thickening, though data remain limited [12].

The turning point in management occurred after initiating empirical anti-TB therapy on day 66 of hospitalization. The patient demonstrated rapid clinical improvement: fever subsided, pleural drainage ceased, oxygen requirements declined, and he was successfully weaned from ventilation. This response further supports the diagnostic utility of ADA and the importance of clinical judgment in initiating therapy.

## Conclusions

In conclusion, diagnosing TPE remains challenging, particularly when microbiological tests are negative. High ADA levels in pleural fluid - especially >70 IU/L combined with a lymphocytic exudate and compatible clinical features - can provide a strong basis for empirical anti-TB therapy. In our patient, this approach led to dramatic improvement and full clinical recovery after a prolonged and complicated hospitalization. The case reinforces the diagnostic and therapeutic role of ADA and supports early treatment initiation in high-probability cases to avoid delayed outcomes. Further studies are needed to refine ADA cutoffs, validate empirical treatment protocols, and clarify the role of fibrinolytics in loculated tuberculous effusions.
